# Functional Study of Novel Bartter’s Syndrome Mutations in ClC-Kb and Rescue by the Accessory Subunit Barttin Toward Personalized Medicine

**DOI:** 10.3389/fphar.2020.00327

**Published:** 2020-03-17

**Authors:** Dalila Sahbani, Bice Strumbo, Silvana Tedeschi, Elena Conte, Giulia Maria Camerino, Elisa Benetti, Giovanni Montini, Gabriella Aceto, Giuseppe Procino, Paola Imbrici, Antonella Liantonio

**Affiliations:** ^1^Department of Pharmacy-Drug Sciences, University of Bari “Aldo Moro”, Bari, Italy; ^2^Laboratory of Medical Genetics, Fondazione IRCCS Ca’ Granda Ospedale Maggiore Policlinico, Milan, Italy; ^3^Nephrology, Dialysis and Transplant Unit, Department of Women’s and Children’s Health, University-Hospital of Padova, Padova, Italy; ^4^Pediatric Nephrology, Dialysis, and Transplant Unit, Fondazione IRCCS Ca’ Granda Ospedale Maggiore Policlinico, Milan, Italy; ^5^Department of Clinical Sciences and Community Health, University of Milan, Milan, Italy; ^6^Pediatric Nephrology Unit, University of Bari, Bari, Italy; ^7^Department of Biosciences, Biotechnologies and Biopharmaceutics, University of Bari “Aldo Moro”, Bari, Italy

**Keywords:** Bartter’s syndrome, barttin, kidney chloride channels, patch clamp, pharmacology

## Abstract

Type III and IV Bartter syndromes (BS) are rare kidney tubulopathies caused by loss-of-function mutations in the *CLCNKB* and *BSND* genes coding respectively for the ClC-Kb chloride channels and accessory subunit barttin. ClC-K channels are expressed in the Henle’s loop, distal convoluted tubule, and cortical collecting ducts of the kidney and contribute to chloride absorption and urine concentration. In our Italian cohort, we identified two new mutations in *CLCNKB*, G167V and G289R, in children affected by BS and previously reported genetic variants, A242E, a chimeric gene and the deletion of the whole *CLCNKB*. All the patients had hypokalemia and metabolic alkalosis, increased serum renin and aldosterone levels and were treated with a symptomatic therapy. In order to define the molecular mechanisms responsible for BS, we co-expressed ClC-Kb wild type and channels with point mutations with barttin in HEK 293 cells and characterized chloride currents through the patch-clamp technique. In addition, we attempted to revert the functional defect caused by BS mutations through barttin overexpression. G167V and A242E channels showed a drastic current reduction compared to wild type, likely suggesting compromised expression of mutant channels at the plasma membrane. Conversely, G289R channel was similar to wild type raising the doubt that an additional mutation in another gene or other mechanisms could account for the clinical phenotype. Interestingly, increasing ClC-K/barttin ratio augmented G167V and A242E mutants’ chloride current amplitudes towards wild type levels. These results confirm a genotype-phenotype correlation in BS and represent a preliminary proof of concept that molecules functioning as molecular chaperones can restore channel function in expression-defective ClC-Kb mutants.

## Introduction

Bartter’s syndromes (BS) are a group of kidney genetic tubulopathies characterized by salt and fluid loss, elevated prostaglandin E2 (PGE2) production, increased renin and aldosterone circulating levels, normal-to-low blood pressure and growth delay (Simon et al., 1997; Birkenhäger et al., 2001; Andrini et al., 2015; Seys et al., 2017; Fulchiero and Seo-Mayer, 2019). Five types of BS have been identified according to the causative genes encoding proteins involved in tubular fluid reabsorption in the thick ascending limb (TAL) of Henle’s loop (Loudon and Fry, 2014; Kleta and Bockenhauer, 2018). In particular, type III BS is caused by loss-of-function mutations of the *CLCNKB* gene encoding for the human ClC-Kb chloride channel, whereas type IV BS is caused either by loss-of-function mutations in *BSND* gene encoding for the ClC-Ks accessory subunit barttin or by simultaneous mutations in *CLCNKB* and *CLCNKA* genes, this latter encoding for the human ClC-Ka isoform. ClC-Ks channels and barttin are expressed in Henle’s loop, distal convoluted tubule, and cortical collecting ducts of the kidney, where they contribute to sodium chloride reabsorption, urine concentration, and consequently blood pressure maintenance (Estévez et al., 2001; Krämer et al., 2008; Fahlke and Fischer, 2010; Barrallo-Gimeno et al., 2015; Pinelli et al., 2016; Jentsch and Pusch, 2018). In the inner ear both ClC-K isoforms contribute to potassium secretion into the endolymph by the marginal cells of the *stria vascularis* (Fahlke and Fischer, 2010). Type III BS is diagnosed in infancy or early childhood and is characterized by a large phenotypic variability, ranging from antenatal BS with polyhydramnios and premature birth, to the less severe Gitelman syndrome (GS) with hypomagnesemia, hypocalciuria, and absence of polyuria (Jeck et al., 2000; Andrini et al., 2015; Seys et al., 2017; Cheng et al., 2017; Walsh et al., 2018). Type IV BS is a more severe variant with additional sensorineural hearing loss (Fulchiero and Seo-Mayer, 2019).

So far more than 50 mutations in the *CLCNKB* gene have been identified in BS individuals, scattered throughout the whole protein sequence including the selectivity filter, barttin binding sites, dimer interface, and C-terminal region (Andrini et al., 2015). Yet, the most common defect is the total *CLCNKB* gene deletion (Simon et al., 1997; Konrad et al., 2000).

The accessory subunit barttin, discovered in 2001, is indispensable for the proper localization of ClC-K channels at the basolateral membrane in renal tubules and inner ear and modulates channels gating (Estévez et al., 2001). Its discovery allowed the functional and pharmacological characterization of both ClC-Kb wild type and mutants in heterologous expression systems (Estévez et al., 2001; Waldegger et al., 2002; Liantonio et al., 2008; Janssen et al., 2009; Liantonio et al., 2012; Imbrici et al., 2014; Gradogna et al., 2014; Liantonio et al., 2016; Imbrici et al., 2017a; Koster et al., 2018). Importantly, barttin mutations associated with BS type IV lead to a drastically reduced functioning of ClC-K channels in heterologous expression systems (Estévez et al., 2001; Hayama et al., 2003; Janssen et al., 2009). Moreover, barttin knock-out and knock-in mice show severely impaired plasma membrane localization of ClC-K channel and reduced transepithelial chloride transport (Rickheit et al., 2008; Nomura et al., 2011). Importantly, a molecule able to increase protein stability and prevent ER degradation, such as 17-allylamino-17-demethoxygeldanamycin (17-AAG), an Hsp90 inhibitor, was reported to mitigate the BS symptoms in barttin R8L knock-in mice by likely enhancing the plasma membrane expression of ClC-K1/mutant barttin channels (Nomura et al., 2013). Barttin seems to interact with hydrophobic residues in helices B and J, at the outer surface of the ClC-K channels, through its short N-terminal and first TM1 helix (Tajima et al., 2007; Wojciechowski et al., 2018a).

Among the ClC-Kb missense mutations functionally studied so far, most resulted in >60% reduction of chloride current primarily due to an alteration of the plasma membrane expression of the channel. Mutations altering channel gating are not frequent in BS (Keck et al., 2013; Andrini et al., 2015; Seys et al., 2017). Reduced channel surface expression can be the consequence of altered synthesis, defective folding, reduced stability and trafficking to the plasma membrane, or increased degradation (Andrini et al., 2015). The manifestation and prognosis of BS depend on the mutation types, and more severe mutations are often associated with younger age at diagnosis, lower serum chloride concentration, and higher urine calcium excretion rate (Seys et al., 2017; Cheng et al., 2017; Yang et al., 2018).

Due to the lack of ClC-Kb channel activators, the pharmacotherapy of BS is based on symptoms relief with limited benefit for patients, poor adherence to medication, and side effects (Imbrici et al., 2016a). As such, BS affected patients are treated with cyclooxygenase inhibitors such as indomethacin to reduce elevated PGE2 levels, potassium and magnesium supplements, and potassium-sparing diuretics to normalize electrolyte balance, and angiotensin-converting enzyme inhibitors or angiotensin receptor blockers to counteract high angiotensin II plasma levels and proteinuria (Alhammadi et al., 2014; Zieg et al., 2016; Yang et al., 2018; Kleta and Bockenhauer, 2018). The possibility to target BS mutations with specific molecular defect with ClC-K channels modulators would be appealing in the perspective to ensure a specific and safer therapy to BS patients (Imbrici et al., 2017b). In this context, drug repositioning could be a pursuable pharmacological strategy (Loizzi et al., 2013; Imbrici et al., 2018; Perucca and Perucca, 2019). A first and essential step in this direction is surely represented by the assessment of the biophysical characterization of mutants for revealing the specific BS channel functional defect.

Here we report five patients affected by severe BS carrying the known mutation A242E and two novel variants G167V and G289R in *CLCNKB*. We provide the functional characterization of the two novel mutations in order to define the molecular mechanism responsible for BS and to assess a useful genotype-phenotype correlation. In addition, we tested the hypothesis that barttin could function as a molecular chaperone by restoring the activity of two expression-defective BS mutants. The results of this study can put the basis for the development of personalized therapeutic options for ClC-K associated diseases.

## Materials and Methods

### Clinical and Genetic Diagnosis

We analyzed five Italian patients that were referred to our clinics due to variable grades of clinical and biochemical manifestations compatible with BS. Clinical examination was specifically conducted to search for BS signs such as growth delay, polydipsia and polyuria, vomiting and anorexia, dehydration, nephrocalcinosis, and the lab parameters associated with renal salt wasting and potassium wasting, impaired reabsorption of chloride, and metabolic alkalosis.

Written informed consent for DNA storage, use for genetic analysis and research purposes as well as for the publication of their cases and identifiable data was obtained from all the patients and relatives, as required by the Ethical Committee of the University of Milan and in accordance with the Declaration of Helsinki. Genomic DNA was extracted from peripheral blood using a QIASymphony AS automated DNA extractor (QIAGEN, Germany). The 19 coding exons and adjacent intronic sequences of the *CLCNKB* gene were amplified by PCR, sequenced with BigDye Terminator v1.1 cycle sequencing kits, and run on an automated ABI PRISM 3130*xl* Genetic Analyzer (Perkin Elmer Applied Biosystems, Foster City, CA), and compared to Genbank sequence NM_000085.4. *CLCNKB* primers sequences are listed in the **Supplemental Table 1**. The missense mutations identified in patients were searched for in 200 Italian control chromosomes and their frequency was checked on The Exome Aggregation Consortium browser (ExAC) (http://exac.broadinstitute.org/). The SALSA MLPA P266 *CLCNKB* Kit (MRC Holland, Amsterdam, The Netherlands) was used for CNVs detection. Two additional genes (*SLC12A3* and *MAGED2*) were sequenced for patient V and CNVs detection in the *SLC12A3* gene was performed by SALSA MLPA P136 Gitelman syndrome Kit (MRC Holland, 161 Amsterdam, The Netherlands).

Variant interpretation was made according to the ACMG guidelines (Richards et al., 2015). *In silico* predictions were performed with SIFT (http://www.Blocks.fhcrc.org/sift/SIFT.html), PolyPhen-2 (http://genetics.bwh.harvard.edu/pph/), Combined Annotation Dependent Depletion (CADD score) (https://cadd.gs.washington.edu/), Mutpred2 (http://mutpred.mutdb.org/about.html), and Mutation Taster (http://www.mutationtaster.org/documentation.html).

### ClC-Kb Mutagenesis, Expression, and Electrophysiology

Mutations were introduced into the plasmid pcDNA3.1-hClC-Kb using the Quickchange™ site-directed mutagenesis kit (Agilent, Santa Clara, CA, USA), as previously described (Imbrici et al., 2016b). The complete coding region of the cDNA was sequenced to exclude polymerase errors. Human ClC-Ks and Y98A barttin cDNAs (courtesy of Professor Michael Pusch and Professor Al George Jr) were subcloned in the pcDNA3 vector. HEK293 cells in a 100 mm dish were transiently transfected with plasmid cDNAs encoding ClC-Kb (5 µg), wild-type or BS mutants, and accessory subunit barttin in a 1:1, 1:2, 1:3, and 1:4 weight ratio (5, 10, 15, or 20 µg), using a Ca^2+^ phosphate precipitation method. For the identification of transfected HEK293 cells, a plasmid encoding the CD8 antigen was co-transfected. The transfected cells were identified by microbeads coated with anti-CD8 antibodies (Dynabeads M-450 CD8; Dynal, Great Neck, NY) and were used for electrophysiological recordings. Patch clamp experiments were performed typically one day after transient transfection (Imbrici et al., 2014). Whole-cell patch-clamp recordings were performed using an Axopatch 200B amplifier (Molecular Devices, Sunnyvale, CA). Pipettes were pulled from borosilicate glass (Harvard Apparatus, Holliston, MA) and had resistances of 2.2 to 3.2 MΩ. The extracellular solution contained 140 mM NaCl, 4 mM KCl, 2 mM CaCl_2_, 1 mM MgCl_2_, and 5 mM HEPES, whereas the pipette solution contained 120 mM NaCl, 2 mM MgCl_2_, 5 mM EGTA, and 10 mM HEPES. Both solutions were adjusted to pH 7.4 with NaOH. Patch recordings for ClC-Kb were obtained by stepping the holding potential from 0 mV to various test potentials from −120 to +100 mV for 400 ms. Pulses ended with a tail pulse to −80 mV for 200 ms. As a control, we routinely applied a solution containing 100 mM I^-^ that blocks currents carried by ClC-Kb channels but not endogenous currents (Imbrici et al., 2014). Current traces at each potential were filtered at 1 kHz with a four-pole low-pass Bessel filter and acquired at 5 kHz with pClamp10 program (Axon Instruments, Sunnyvale CA, USA). MG-132 and 17-AAG were purchased from Sigma.

### Confocal Microscopy

HEK 293 cells were grown on glass coverslips, transfected with cDNA encoding EGFP–ClC-Kb WT, G167V and A242E (5 µg) and untagged barttin, and subjected to immunofluorescence 48 h after transfection, when they reached the confluency. The ratio between ClC-Kb and barttin was either 1:1 or 1:3 (5 or 15 µg) to mimic a presumed physiological condition in the first case and an overexpression of barttin in the latter. Transfection was performed using Lipofectamine™ 2000 reagent (www.thermofisher.com), according to the manufacturer’s protocol. Plasma membrane was stained with wheat germ Agglutinin, Alexa Fluor™ 555 Conjugate (WGA-555; www.thermofisher.com) in Hank’s balanced salt solution (HBSS) for 30 min at 37°C at a concentration of 5 µg/ml. Cell coverslips were mounted on glass microscopy slides and observed on a Nikon Eclipse TE 2000-U fluorescent microscope equipped with a 40X/1.30 N.A. fluor objective (Nikon) and a spinning-disk confocal setup (assembled by CRISEL Instruments, https://www.crisel-instruments.it). EGFP-ClC-Kb fluorescence was excited with a green laser (SVL-473-0200 at 473 nm of excitation wavelength) and recorded at 520 nm emission wavelength. Wheat germ agglutinin AlexaFluor™ 555 fluorescence was excited using a mercury lamp light, selecting an excitation at 555 nm on the excitation filter wheel and emission at 585 nm on the emission filter wheel. Excitation light was projected through 1000 pinholes (Ø 70 µm) using a CREST CARVII™ spinning disk. Both fluorophores emission wavelengths were collected by a Photometrics Cool Snap HQ camera (1392 × 1040 imaging pixels) and digitalized with the MetaMorph^®^ software (www.moleculardevices.com).

### Biotinylation of Cell Surface Proteins

Cell surface biotinylation was carried out with the Pierce Cell Surface Protein Isolation Kit (Pierce, Waltham, MA, USA). HEK 293 cells were transfected with cDNAs coding for wild type ClC-Kb, A242E, G167V (5 µg), and barttin at 1:1 or 1:3 ratio (5 or 15 µg), using a Ca^2+^ phosphate precipitation method. On day after transfection, cell surface proteins were labeled with sulfosuccinimidyl-2-(biotinamido)ethyl-1,3-dithiopropionate (Sulfo-NHS-SS-biotin). Briefly, cells were washed with ice cold PBS twice, and Sulfo-NHS-SS-biotin was added and incubated at 4°C with constant rotation for 30 min. Excess biotin was quenched with quenching solution. Cells were treated with lysis buffer and centrifuged at 10,000 *g* for 2 min at 4°C. Clear supernatant was reacted with immobilized NeutrAvidin gel slurry in columns to isolate surface proteins. Columns were washed and protein eluted in sample buffer containing DTT. Surface proteins were separated on a SDS-PAGE gel and the samples (10 µg) were analyzed by western blotting using a monoclonal anti-ClC-Kb antibody (Abcam, ab66460). Filters were also immunoblotted with β-actin monoclonal antibody (Sigma) as control.

### Data and Statistical Analysis

Patch clamp recordings were analyzed off-line by using pClamp 10.3 (Axon Instruments, Sunnyvale CA, USA) and Sigma Plot Software (Systat Software GmbH, Germany). Statistical analysis was performed using Student’s t-test, with p < 0.05 or less considered as significant. Results are reported as mean ± SEM from the indicated number of cells in each experiment.

## Results

### Clinical and Genetic Diagnosis

Here we report five Italian patients with genetically and/or clinically defined type III BS. The clinical phenotypes of the BS patients and corresponding *CLCNKB* mutations are summarized in **Table 1**.

**Table 1:** Clinical features at presentation of the examined patients with Type 3 BS.

	cDNA (NM_000085.4) and aminoacidic variation	Sex and age at onset	Heterozygous/homozygous	Serum K^+^ (mmol/L)	Serum Mg^2+^ (mg/dl)	Serum HCO_3_^-^ (mmol/L)	Serum renin (ng/ml/h)	Serum aldosterone (pg/ml)	Serum creatinine (mg/dl)	Urinary Ca^2+^ (mg/dl)	Clinical parameters
**Patient I**	c.500G > T p.Gly167Val	M, 2 years old	Heterozygous with *CLCNKB* deletion	2,3	2,8	33,0	16,26	476	0,3	3,2	Growth delay, hematuria, polydipsia, polyuria, nephrocalcinosis
**Patient II**	c.725C > A p.Ala242Glu	F, 6 months	Heterozygous with *CLCNKB* deletion	2,8	2,3	55,6	N.D.	800	0,2	7,84	Growth delay, polyhydramnios, asthenia
**Patient III**	c.725C > A p.Ala242Glu	F, 5 months	Heterozygous with CLCNKA/CLCNKB Chimeric gene^§^	3,0	1,8	55,0	264,6	30.9	1,34	0.6	Slight reduction of weight growth, vomit, anorexia, dehydration
**Patient IV**	c.725C > A p.Ala242Glu	F, 23 years old	Homozygous	1,8	1,8	26,5	N.D.	N.D.	1,3	N.D.	Asthenia, polydipsia, cramps, tingling, abdominal pain, polyuria, heart problems, tetany, hyperpyrexia
**Patient V**	c.865G > C p.Gly289Arg	M, 5 months	Homozygous	2,3	0,92	44,1	30	523,7	0,84	0,02	Slowing growth from the second month, vomit, polyuria, anorexia

^§^Deletion spanning from CLCNKA Exon 7 to CLCNKB Exon 6 included; HGVS nomenclature: chr1:g.(16353109_16353191) _(16374915_16374998)del.

The G167V mutation (c.500G > T) has been detected in a 2-year-old child with growth delay, polydipsia, polyuria, hyperaldosteronism, hypokalemia, and nephrocalcinosis (Patient I). The mutation is compound heterozygous with the deletion of the *CLCNKB* gene. The child is under KCl and NaCl supplements.

The A242E mutation (c.725C > A) has been identified in three patients (Patients II-III-IV). This amino acid variation had already been described (Bettinelli et al., 2007). Two female carriers presented early onset BS at the age of 5 and 6 months. Both children showed severe phenotypes with growth delay, vomit, anorexia and dehydration, high levels of renin and aldosterone, and hypokalemia. In the 6 months girl (Patient II) the mutation is heterozygous with the deletion of the entire *CLCNKB* gene. She is treated with KCl supplements. In the 5 months girl (Patient III), born at term, the mutation is associated with a heterozygous deletion spanning from *CLCNKA* exon 7 to *CLCNKB* exon 6 included (chimeric gene very common in the Italian Puglia region and in Albania, suggesting a common ancestor, data not shown). She is treated with potassium sparing diuretic canrenone and KCl supplement, calcium carbonate, ranitidine, and vitamin D. The third carrier of A242E mutation (Patient IV), who manifested BS at 23 years old, is homozygous for the mutation. This woman presented with a Gitelman phenotype at the onset but was negative for mutations in *SLC12A3*. She presents with hypokalemia and hypocalciuria, asthenia, polydipsia, cramps, tingling, abdominal pain, polyuria, heart problems, tetany, hyperpyrexia, gastroesophageal reflux, recurrent proteinuria. She was initially (1986) administered indomethacin then discontinued in 2005 and replaced with angiotensin-converting enzyme inhibitor enalapril and potassium and magnesium citrate. Since 2014 she is under atorvastatin, xanthine-oxidase inhibitor febuxostat, potassium and magnesium citrate, and enalapril.

Finally, the homozygous G289R mutation (c.865G> C) was identified in a boy, born at term after an uncomplicated pregnancy (no polyhydramnios), who presented at the age of 5 months with vomit, anorexia with feeding difficulties, and polyuria (Patient V). This patient was screened for two other genes, *SLC12A3* and *MAGED2*, with negative results. Growth chart displayed a regular weight gain until the second month of life and then a drop from 50th to 3rd percentile. Remarkable blood and urine findings included hypokalemia, hypomagnesemia, hypochloremic metabolic alkalosis, hyperuricemia, high blood levels of renin and aldosterone, and hypocalciuria. Potassium citrate and NaCl supplementation was started, as well as nasogastric tube feeding (maintained until 15 months of age). At last follow up, he was a healthy-appearing 10 years old boy, with normal growth (weight in the 50^th^–75^th^ percentile and height in the 25^th^ percentile). Blood and urine analyses were normal. Therapy still includes potassium and sodium chloride supplementation and the potassium-sparing agent spironolactone.

The missense mutations identified in our patients were not found in 200 Italian control chromosomes.

### Functional Characterization of ClC-Kb Mutant Channels

**Figure 1:**
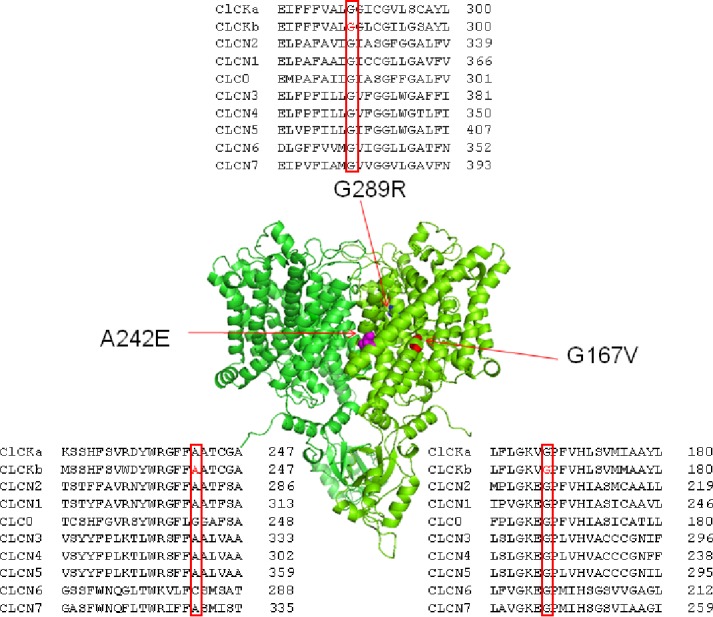
Structure and alignment of ClC-Kb channel. Three dimensional representation of the ClC-Kb channel modeled upon the cryo-electron microscopy structure of the bovine ClC-K (PDB id: 5TQQ; Park et al., 2017) showing the localization of the BS mutations. The insets show the amino acids alignment of CLC proteins highlighting the position of the BS mutations.

Mutated residues G167V, A242E, and G289R were located on critical helices for channel function (F, I, and J respectively) and were conserved within CLC family (**Figure 1**). To test whether the two newly identified mutations, G167V and G289R, were responsible for the occurrence of BS in the affected carriers, we expressed the same amount (5 μg) of wild type and mutant ClC-Kb channels cDNA together with barttin (1:1 ratio) in HEK 293 cells and recorded chloride currents through whole-cell patch-clamp. The mutation A242E has been previously identified and characterized (Bettinelli et al., 2007; Cheng et al., 2017); in HEK cells A242E mutant channels showed poor membrane expression (9% of the wild type) and markedly reduced current levels (Cheng et al., 2017).

**Figure 2:**
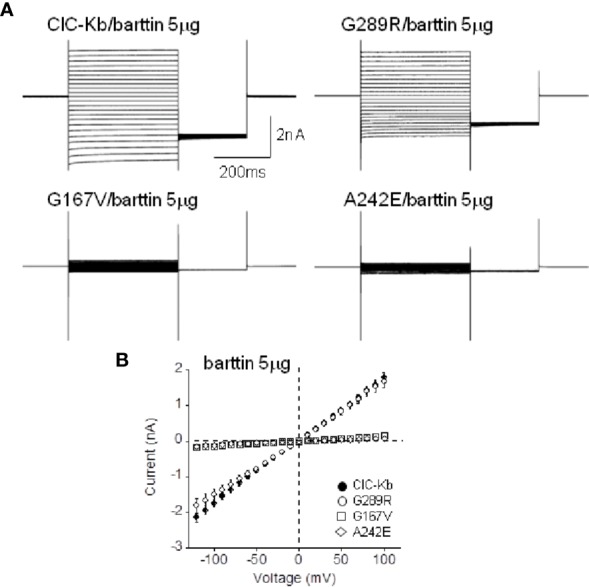
Functional characterization of ClC-Kb mutants expressed in HEK 293 cells. **(A)** Representative chloride current traces of ClC-Kb wild type, G167V, G289R, and A242E channels (5 µg) co-expressed with equal amount of barttin (1:1 ratio) in HEK 293 cells. **(B)** IV plot showing the mean current amplitude of the indicated channels as a function of membrane potential. Data are mean ± SE; n = 11–19 cells.

G167V is located in helix F and is predicted to be pathogenic (MutPred2 score 0.835). Consistently, mutant channel current was 10% compared to that of wild type (0.08 ± 0.01 nA vs 1.05 ± 0.08 nA, n = 16–19; **Figures 2A, B**). In contrast, the G289R mutation (MutPred2 score of 0.876) in helix J, produced chloride currents similar to wild type (1.01 ± 0.10 nA vs 1.05 ± 0.08 nA, n = 11–19; **Figures 2A, B**). We confirmed that, also in our experimental conditions, mutant A242E channels (MutPred2 score 0.803) significantly reduced ClC-Kb chloride current by 90% (0.05 ± 0.01 nA vs 1.05 ± 0.08 nA, n = 14–19; **Figures 2A, B**).

The results obtained with G167V channels lead us to hypothesize that the almost total absence of chloride current produced by this mutant might be at least in part due to the strongly reduced expression in the membrane, similarly to A242E (Cheng et al., 2017).

**Figure 3:**
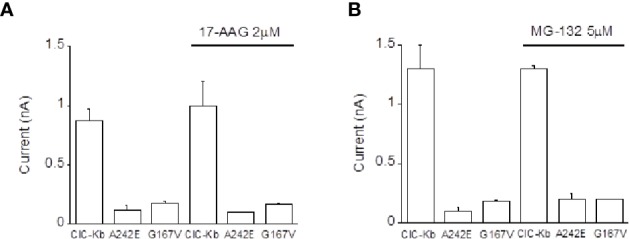
Effect of Hsp90 and proteasome inhibition on ClC-Kb mutants expressed in HEK 293 cells. Bar graphs showing the effect of incubation of HEK 293 expressing ClC-Kb wild type, G167V and A242E channels with **(A)** 17-AAG (16 h) and with **(B)** MG-132 (12 h). ClC-Kb subunits were co-expressed with 5 µg of barttin. Data are mean ± SE; n = 6–9 cells.

In the attempt to define the molecular mechanisms underlying the observed BS mutants reduced expression, we incubated ClC-K-expressing HEK cells with 17-AAG, an Hsp90 inhibitor and with MG-132, a potent proteasome inhibitor previously found to rescue one ClC-1 mutant *in vitro* (Lee et al., 2013). Neither compounds succeeded in rescuing the activity of A242E and G167V channels (**Figures 3A, B**). Thus, these data allowed us to exclude the involvement of these investigated pathway in mutants channels expression.

### Effect of Barttin Overexpression on ClC-Kb Wild Type and Mutant Channels Current Amplitude and Membrane Localization

ClC-K channels need the accessory subunit barttin for surface expression and full activity (Estévez et al., 2001). For this reason we tested the hypothesis that an increased amount of co-expressed accessory subunit (10, 15, and 20 µg corresponding to 1:2, 1:3, 1:4 ClC-Kb:barttin ratio, respectively) could facilitate the transport of the A242E and G167V mutant channels (5 µg) to the membrane and rescue their functional phenotype.

**Figure 4:**
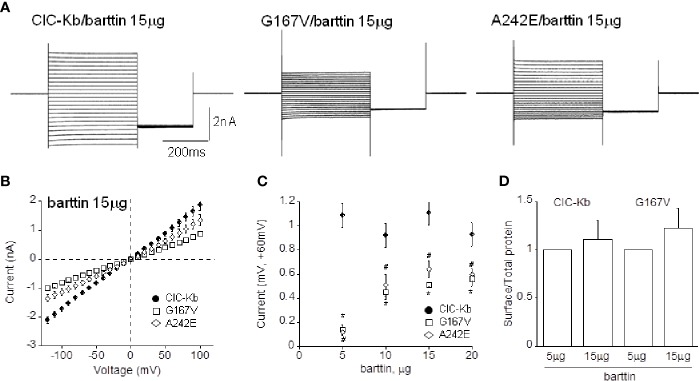
Effect of barttin overexpression on ClC-Kb wild type, G167V and A242E current amplitude. **(A)** Representative chloride current traces of ClC-Kb wild type, G167V, G289R, and A242E channels (5 µg) co-expressed with 15 µg barttin (1:3 ratio) in HEK 293 cells. **(B)** IV plot showing the mean current amplitude as a function of membrane potential of the indicated channels co-expressed with 15 µg barttin. **(C)** Chloride current measured at +60 mV as a function of the amount of co-expressed barttin for ClC-Kb WT, G167V, and A242E. Data are mean ± SE; n = 6–19. p < 0.05 for G167V (*) and A242E (#) compared with the respective ClC-Kb wild type. **(D)** Quantification of the surface expression efficiency for ClC-Kb and G167V channels co-expressed with 5 or 15 µg of barttin calculated by dividing surface protein density to the respective total protein density. The mean surface density of ClC-Kb and G167V channels co-expressed with 15 µg of barttin was normalized to the corresponding density of the same channels co-expressed with 5 µg of barttin. Data are mean ± SE of n = 3 experiments.

Augmenting barttin did not affect the amplitude of wild type chloride currents (1.08 ± 0.08 nA, 1.07 ± 0.10 nA, 0.88 ± 0.09 nA corresponding to 1:2, 1:3, 1:4 ratio, respectively, n = 6–15; **Figures 4A–C**), suggesting that 5 µg of the accessory subunit already ensured maximal protein expression.

Ten and 15 µg of barttin increased A242E current by 4-and 6-fold compared with that measured with 5 µg of barttin; A242E current became then 40% and 60% of wild type current value (0.40 ± 0.07 nA, 0.64 ± 0.07 nA at +60mV, respectively, n = 6–10; **Figures 4A–C**). Under the same experimental conditions (10 and 15 µg of barttin), G167V current increased by 5-fold compared with that measured with 5 µg of barttin, becoming 50% of wild type (0.51 ± 0.06 nA and 0.50 ± 0.02 nA, respectively, n = 6–16; **Figures 4A–C**). Therefore, tripling the amount of barttin boosted mutants chloride currents towards wild type level despite never totally equaling it. An additional increase of barttin (20 µg) did not induce a further rise of mutant channels currents, suggesting that current amplitude reached the steady-state in this experimental condition (0.60 ± 0.052 nA and 0.56 ± 0.060 nA, for A242E and G167V respectively; **Figure 4C**).

To address whether the overexpression of barttin increased chloride currents by promoting the surface expression of the mutant proteins, we performed protein biotinylation assay on HEK 293 cells expressing WT and G167V channels co-expressed with barttin in 1:1 and 1:3 (5 or 15 µg). **Figure 4D** shows that 15 µg barttin improved the relative surface density of G167V channels, suggesting that enhanced trafficking of the mutant protein may account for the observed increase of current level (**Figure 4D**, **Supplemental Figure 1**).

**Figure 5:**
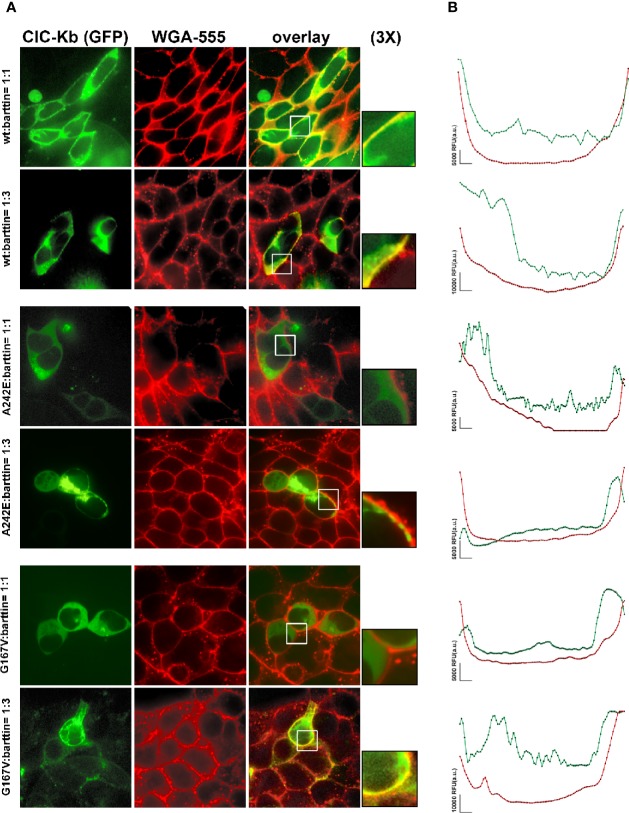
Effect of barttin overexpression on ClC-Kb wild type, G167V and A242E membrane localization. **(A)** Immunofluorescence confocal microscopy analysis of the plasma membrane marker WGA-555 (red signal) and EGFP-ClC-Kb WT, A242E and G167V (green signal), transfected with the same amount of barttin (5 µg: 5 µg, 1:1 ratio, upper panels) or overexpressing barttin (5 µg: 15 µg, 1:3 ratio, lower panels) in HEK 293 cells grown on coverslips. The overlay column reports the co-localization of the two fluorescence signals. Areas indicated by the white boxes are magnified three times. **(B)** Representative quantitative analysis of EGFP-ClC-Kb WT, A242E and G167V (green signal), and WGA (red signal) fluorescence distribution between plasma membrane and cytosol along a fixed yellow line. Peaks of ClC-Kb overlapped with peaks of WGA in cells transfected with either ClC-Kb WT or with both A242E and G167V mutants co-transfected with an excess of barttin. Similar results were obtained when the analysis was repeated in 10 transfected cells in four different fields using MetaMorph as imaging software. The vertical bar indicates 5,000 RFU (a.u.). WGA, Wheat Germ Agglutinin.

To further support the hypothesis that augmenting barttin might rescue ClC-K channels surface expression and current amplitude, we also performed spinning-disk confocal microscopy analysis to check the plasma membrane localization of ClC-Kb mutants compared with the wild type channel in HEK 293 cells. We used wheat germ agglutinin AlexaFluor™555 to stain cells plasma membranes and evaluated the co-localization with the GFP-tagged ClC-Kb. As shown in **Figure 5**, wild type ClC-Kb (green), regardless of the amount of co-transfected barttin (1:1 or 1:3), was mainly expressed at the plasma membrane as shown by the high degree of co-localization with WGA-555 (see yellow signal in the overlay column and in the 3X magnified boxes). Both A242E and G176V ClC-Kb mutants, when transfected at 1:1 ratio with barttin, were mainly localized in the cells cytoplasm and apparently failed to co-localize with the plasma membrane marker, as shown by the absence of overlapping between the green and red fluorescence in the merge column even at further 3× magnification of the pictures. Interestingly, overexpression of barttin (1:3) partially rescued plasma membrane expression of both mutants, as indicated by the increased level of co-localization with the plasma membrane marker WGA-555 (**Figure 5**, **Supplemental Figure 2**).

## Discussion

In this study we provide the functional characterization of two novel ClC-Kb mutations associated with BS phenotypes in children, G167V and G289R, and demonstrate that the accessory subunit barttin can partially restore G167V and A242E channels function.

### Genotype-Phenotype Correlation

Hypochloremia, hypokalemia, metabolic alkalosis, and growth retardation are the most common manifestations of type III BS observed in the G167V (Patient I) and A242E carriers (Patients II-III). The functional characterization of A242E and G167V channels suggests that the degree of chloride current reduction caused by these mutations clearly correlates with the severity of the associated clinical phenotypes (younger age at diagnosis and lower electrolyte levels; **Table 1**).

As shown for other BS mutations in ClC-Kb, our microscopy, biochemical, and electrophysiological data show that mutations affecting the dimerization interface (A242E) or the channel pore (G167V) cause severe channel dysfunctions. In addition, the observed marked reduction in current amplitude shown by G167V and A242E channels is consistent with a reduced number of active channels in the plasma membrane more than to channel closure due to a biophysical defect (Andrini et al., 2014; Andrini et al., 2015; Cheng et al., 2017; Seys et al., 2017). We also demonstrated that inhibiting Hsp90 or proteasome did not cause any rescue of A242E and G167V mutant currents, thus suggesting that these pathways are not involved in the altered expression of the mutants under investigation.

G167 is well-conserved in the CLC family and is located very close to K165 and V166 in the F helix N-terminus, a structural region known for its predominant role in the chloride pathway (Park et al., 2017; **Figure 1**). It is high likely that the replacement of the small glycine at position 167 with the bulkier (longer side chain) valine could change the orientation of the V166 side-chain, thus altering channel folding and stability and, in turn, the sorting to the plasma membrane. Confocal microscopy and biochemical data support this idea, although we cannot exclude the occurrence of a minor biophysical defect. In particular, MD simulations using a model of human ClC-Kb build upon the 3D structure of the bovine channel, suggest that the rotation of V166 side chain together with the slight tilting of helix F are mainly responsible for the opening of the pore and release (or binding) of chloride ion at S_ext_ position (Louet et al., 2017).

Of the three carriers of A242E mutations, the homozygous 23 years old woman (Patient IV) presents with a milder phenotype with respect to the 5 and 6 months infants (Patients II and III). The compound heterozygous partial and total deletion of *CLCNKB* in these two latter cases may cause a more severe and irreversible clinical phenotype than in the homozygous A242E carrier. The A242E mutation is placed in helix I at the dimer interface of two ClC-Kb subunits. The mutation consists in the replacement of a neutral alanine with a large and negatively charged glutamate. The presence of this charged residue may modify the strong interaction with residues found in the vicinity of residue 242, such as D49, K196, and R238, and with residues of the counterpart subunit, thus negatively affecting the stability of the dimeric protein (Louet et al., 2017; Cheng et al., 2017). Close to A242, the mutant channel G246R shows very reduced current and protein amount suggesting similar destabilization of the nascent channel (Keck et al., 2013).

The mutation G289R was identified in a 5 months boy with growth delay, vomiting, hypocalciuria, and hypomagnesemia (Patient V). In G289R channels, the hydrophobic glycine is replaced by a charged residue in helix J, a putative barttin binding site. Despite predicted to be of uncertain significance (VUS) according to ACMG (American College of Medical Genetics and Genomics) criteria (the variant is not reported in ExAC database), all *in silico* predictors scores such as MutPred2 (0.876), Mutation Taster (Disease causing), SIFT (Damaging), Polyphen (probably damaging), and CADD (29.2) agree in defining the variant as pathogenic. Even though these predictions are likely due to a supposed impaired barttin-channel interaction, the biophysical phenotype of G289R mutant channels does not easily explain the clinical phenotype of the affected patient, thus putting in doubt the pathogenicity of the mutation. Similarly, BS mutations in the same helix, G296D and S297R, caused a mild decrease in current levels and protein expression (Cheng et al., 2017). In the G289R carrier serum magnesium and urinary calcium levels were low, suggesting a GS-like phenotype; the *SLC12A3* gene, encoding for the thiazide-sensitive sodium-chloride co-transporter (NCC), was thus screened but no gene variant was detected. Recently, loss-of-function mutations in *MAGED2* (melanoma-associated antigen D2) gene have been associated with a severe but transient clinical form of BS. These mutations seem to cause inappropriate expression of the sodium–chloride transporters NKCC2 and NCC (Legrand et al., 2018; Laghmani et al., 2016). Considering that the G289R carrier appeared healthy, with normal growth, blood, and urine values at follow-up, and given the functional test result that questioned the pathogenicity of this variant, only this patient was further screened for *MAGED2* gene, in order to exclude that causative mutations in other genes possibly implicated in the disease were undetected. Despite the negative results of the two additional genes analyzed, we cannot definitely exclude that G289R is a causative mutation acting with an undefined pathological mechanism (other than a reduced membrane expression leading to a reduced chloride current), especially considering the agreed *in silico* prediction and a “Variant of Unknown Significance” classification according to Clinvar.

### Perspectives for BS Therapy

According to our electrophysiological, biochemical, and imaging findings, the overexpression of the accessory subunit barttin succeeded in restoring at least in part the function of the expression-defective mutants G167V and A242E towards wild type levels. It is widely demonstrated that barttin is able to improve the stability of ClC-K channels by promoting complex glycosylation of the nascent proteins, thus facilitating effective exit from the ER and Golgi and sorting to the plasma membrane, besides modulating channel gating (Estévez et al., 2001; Waldegger et al., 2002; Hayama et al., 2003; Scholl et al., 2006; Janssen et al., 2009; Fischer et al., 2010; Wojciechowski et al., 2018a; Wojciechowski et al., 2018b). We could therefore likely infer that the same mechanism underlies BS mutant channels rescue. Particularly, in the cases described here, a higher barttin amount might protect G167V and A242E misfolded or destabilized mutants from premature degradation, leading to increased chloride current levels. This increase of chloride current obtained upon barttin overexpression raises the intriguing possibility that molecules able to favor the selective ClC-K protein stabilization and activity, could be explored to counteract the expression deficiency of some BS mutants.

Defective expression of ion channels is quite common in loss-of-function channelopathies (Imbrici et al., 2016a; Terragni et al., 2017). Recent pharmacological approaches to treat channelopathies are therefore focusing on pharmacochaperones, lipophilic compounds able to cross the cell membrane and reach their target proteins within the cell, working as molecular scaffolds to stabilize misfolded proteins and promote correct trafficking to their site of action (Hou et al., 2018). Successful attempts to correct trafficking defects using molecular chaperones have been reported for CFTR mutants causing cystic fibrosis or lysosomal storage disorders (Zegarra-Moran and Galietta, 2017; Platt, 2018). Importantly, in our study, upon barttin overexpression, G167V and A242E currents reach similar amplitudes to those reported for ClC-Ks co-expressed with two different barttin mutations that reduce chloride current to a level sufficient to impair hearing but not renal function (Riazuddin et al., 2009; Tan et al., 2017). These evidences add support to the idea that the recovery, even partial, of ClC-Kb impaired function could be a correct therapeutic strategy to treat BS. Such an approach could offer a unique opportunity to the pharmacotherapy of BS, switching from a symptomatic therapy to a personalized defect-targeted cure, using molecules targeting the specific channel defect induced by a mutation (Terragni et al., 2017; Imbrici et al., 2017b).

## Data Availability Statement

The datasets generated for this study can be found in the LOVD, p.Gly167Val: https://databases.lovd.nl/shared/variants/0000644244#00005238, p.Gly289Arg: https://databases.lovd.nl/shared/variants/0000644249#00005238, p.Ala242Glu: https://databases.lovd.nl/shared/variants/0000644245#00005238.

## Author Contributions

PI and AL conceived, coordinated the study, and wrote the paper. GM, GA, and EB performed the clinical diagnosis. BS and ST performed the genetic diagnosis. DS performed mutagenesis and electrophysiologic experiments. GP performed the confocal microscopy experiments. EC and GC performed the biochemical experiments. All authors approved the final version of the manuscript.

## Funding

This work was supported by PRIN 2017 grant to AL and by Fondi Ateneo 2015-16 to PI.

## Conflict of Interest

The authors declare that the research was conducted in the absence of any commercial or financial relationships that could be construed as a potential conflict of interest.

The handling editor declared a past co-authorship with one of the authors GM.
