# Supplementary figures and images for: Functional Study of Novel Bartter’s Syndrome Mutations in ClC-Kb and Rescue by the Accessory Subunit Barttin Toward Personalized Medicine
Source: Front Pharmacol. 2020 Mar 17;11:327. doi: 10.3389/fphar.2020.00327 (PMC7092721; doi:10.3389/fphar.2020.00327)

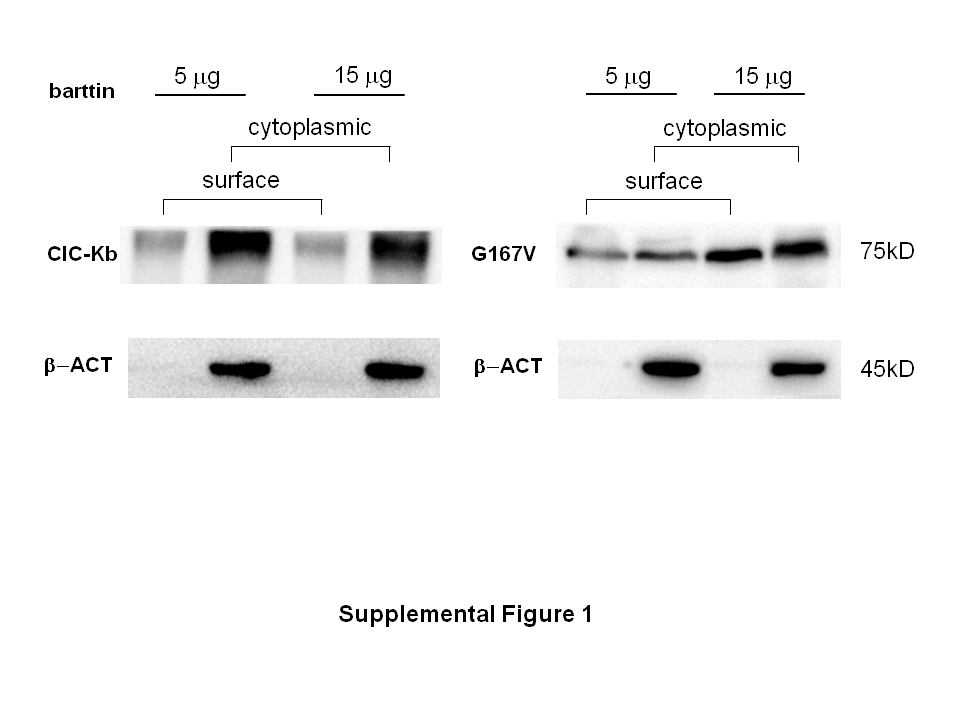

Supplement: Supplementary file 1 [file Image_1.tif]

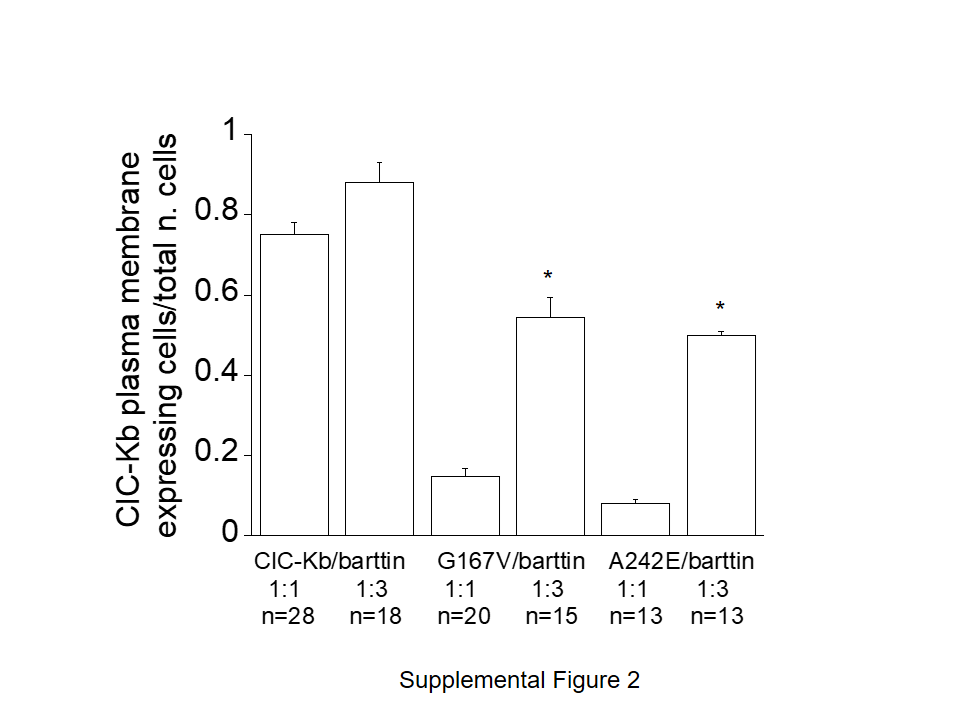

Supplement: Supplementary file 2 [file Image_2.tif]
