# Supplementary material for: Functional Study of Novel Bartter’s Syndrome Mutations in ClC-Kb and Rescue by the Accessory Subunit Barttin Toward Personalized Medicine
Source: Front Pharmacol. 2020 Mar 17;11:327. doi: 10.3389/fphar.2020.00327 (PMC7092721; doi:10.3389/fphar.2020.00327)
Supplement: Supplementary file 3 [file Table_1.docx]

**Supplemental Table 1.** *CLCNKB* Sanger sequencing primers. Refseq: NM_000085

| **Amplicon** | **Forward** | **Reverse** |
| --- | --- | --- |
| 1 | ACCGCGGTCCCTCCCTCTAT | GATGTCCTGAGTGGTCCTCCA |
| 2-3 | GTATACCACCAAGCTCCATCCC | AGTGGGGACTGGCGTAGCGAC |
| 4-6 | TGATCTGGCGAGATCGTAATGTG | GGGTCATACGTGGATTTCAAACC |
| 7-9 | GGTTTGAAATCCACGTATGACCC | AGCTCGCTGAGAGGTCCCCAG |
| 10-11 | CAGCCCTAGAGCCCACCCATC | CAGCTCTGTGCACACCTGGGG |
| 12-13 | TGTCCACGCCTTGCCCAGCAG | CACGACATTGCCCACGCAGCAG |
| 14 | GTGCCAGCCTTGCCCTAACATG | GTCCGAGGTCAGCTACGGTGGC |
| 15 | AGGCTGCTGGGTAAAACAGG | GGGACTCATCTGGGGAATGC |
| 16-17 | GAACAGTTCTTGGCTAAGTAGGTG | CCAGAGGCCTCATGTGTCACA |
| 18 | GGGCACCTTCTACCCTCCAGTG | GTCTTCTCAGGCATAGGTTCCCTG |
| 19 | CAGAAACCACCCTTAGGGGA | AGGGTCTCAGCCCAACCTC |
